# The future of breast ultrasonography through non-mass lesions

**DOI:** 10.1007/s10396-023-01381-0

**Published:** 2023-11-02

**Authors:** Takayoshi Uematsu

**Affiliations:** Department of Breast Imaging and Breast Intervention Radiology, Department of Clinical Physiology, Shizuoka Cancer Center Hospital, Nagaizumi, Japan

I thank Dr. Kurt for her interest in my article [1] and her positive comments on non-mass lesions on breast ultrasonography.

Breast screening ultrasonography should be the cornerstone of a next-generation population-based breast cancer screening program for women with dense breasts [2, 3]. Women with dense breasts seem to benefit little from screening mammography alone due to low mammographic sensitivity. More-effective breast cancer screening modalities are needed. Breast ultrasonography has been proposed as a possible supplemental modality in breast cancer screening given mammography’s low sensitivity related to masking. Ultrasonography is an inexpensive, convenient, readily available, and radiation-free breast imaging modality that also avoids the need for breast compression. Furthermore, a meta-analysis comparing mammography alone with supplemental screening ultrasonography reported an approximately 40% increase in the cancer detection rate for women with dense breasts [4]. In addition, J-START, the world’s first large-scale, randomized, controlled trial of supplemental screening ultrasonography in women 40–49 years of age, demonstrated that supplemental ultrasonography not only increased the sensitivity for and the detection rate of early invasive cancers in the intervention group compared with the control group but also lowered the rate of interval cancers[5]. Although mortality rate is the most important parameter for evaluating the efficacy of supplemental screening ultrasonography, preliminary results from J-START are essential in guiding women with dense breasts in their choice of personalized breast cancer screening.

However, ultrasonography is highly operator-dependent and thus could lead to many false-positive results for women with dense breasts. Its positive predictive value might, therefore, be lower, and its specificity limited. Quality control will be particularly important to help minimize screening-associated harms. In addition, achieving familiarity with breast ultrasonography techniques based on histopathologic anatomic knowledge will be critical in detecting subtle abnormal lesions such as ductal carcinoma in situ, which usually manifests non-mass lesions on breast ultrasonography [1, 2]. Therefore, the standardized terminology for describing non-mass lesions detected on breast ultrasonography will be important.

Superb microvascular imaging allows visualization of microvascular blood flow without the need for contrast agents [6, 7]. Elastography assesses the mechanical properties of tissues, like stiffness or elasticity, to differentiate between malignant and benign lesions, and it could aid in the early detection of breast cancer as tumor tissues are generally stiffer than surrounding healthy tissue, as well as help reduce the number of unnecessary biopsies [6, 8]. I totally agree with Dr. Kurt. Superb microvascular imaging and elastography will additionally characterize non-mass lesions detected on breast ultrasonography without a doubt.

## Data Availability

Data sharing is not applicable to this article as no new data were created or analyzed in this study.
